# Human Development IX: A Model of the Wholeness of Man, His Consciousness, and Collective Consciousness

**DOI:** 10.1100/tsw.2006.258

**Published:** 2006-11-14

**Authors:** Søren Ventegodt, Tyge Dahl Hermansen, Trine Flensborg-Madsen, Erik Rald, Maj Lyck Nielsen, Joav Merrick

**Affiliations:** ^1^ Quality of Life Research Center, Teglgårdstræde 4-8, DK-1452 Copenhagen K, Denmark; ^2^ Research Clinic for Holistic Medicine, Copenhagen, Denmark; ^3^ Nordic School of Holistic Medicine, Copenhagen, Denmark; ^4^ Scandinavian Foundation for Holistic Medicine, Sandvika, Norway; ^5^ Zusman Child Development Center Soroka University Medical Center Ben Gurion University of the Negev, Beer-Sheva, Israel; ^6^ National Institute of Child Health and Human Development, Jerusalem, Israel; ^7^ Office of the Medical Director, Division for Mental Retardation, Ministry of Social Affairs, Jerusalem, Israel

**Keywords:** quality of Life, QOL, holistic biology, theoretic biology, clinical holistic medicine, public health, Denmark

## Abstract

In this paper we look at the rational and the emotional interpretation of reality in the human brain and being, and discuss the representation of the brain-mind (ego), the body-mind (Id), and the outer world in the human wholeness (the I or “soul”). Based on this we discuss a number of factors including the coherence between perception, attention and consciousness, and the relation between thought, fantasies, visions and dreams. We discuss and explain concepts as intent, will, morals and ethics. The Jungian concept of the human collective conscious and unconscious is also analyzed. We also hypothesis on the nature of intuition and consider the source of religious experience of man. These phenomena are explained based on the concept of deep quantum chemistry and infinite dancing fractal spirals making up the energetic backbone of the world. In this paper we consider man as a real wholeness and debate the concepts of subjectivity, consciousness and intent that can be deduced from such a perspective.

## INTRODUCTION

Until now in this series of papers on human development we have been looking at information transmitting interactions at the different levels of the organism. The nature of the information transmitting interactions is unknown, because we do not know the limits of their effect in time and space. Likewise, we cannot with our rational interpretation set this delimitation. The emotional interpretation is far more suitable for this, but unfortunately far from our reason. Epistemologically we here refer to Occam's razor[1,2] and so far maintain for reasons of simplicity that the same principles that affects the lower levels also affects the higher levels. Our conclusion therefore is that the perceptual and conscious life of man may be deduced from a generalization of the effect of the information transmitting interactions to all levels.

In this paper we discuss the representation of the wholeness of the organism and the outer world in the brain. Among all the organ systems the brain is exceptional, because it is specialized to represent the outer world through the sense organs and thereby give a simple interpretation[3] of matter with some or all elements being reduced to the same frame of description as applies to dead things. The representation of the outer world is made up as a long chain of interpretation actions corresponding with the outer world - inasmuch as the motor apparatus is under control of the brain[4]. In Fig. 1 the motor apparatus is drawn below the brain, however the wholeness and the motor apparatus must be thought of as being interacting around the brain. The organism is able to represent the reality (the world) in two ways: 1) Directly through information transmitting interactions, which is the emotional way of representation, where reality is represented in the organism's wholeness[3] or 2) Indirectly through representation in the brain. This is the rational way of representing the outer world by means of the reason[3].

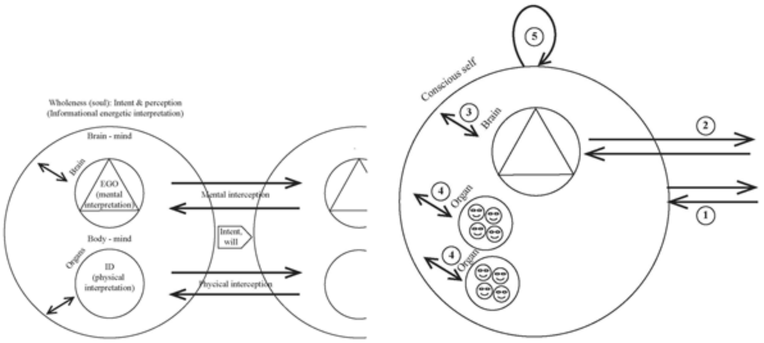

**FIGURE 1.** A model of the wholeness of man, including the functions of attention and will. The representational stages of a human being. The attention function and a survey over the representative possibilities and functional stages. This figure shows a survey of man. Man himself is part of a larger social system, to which it through interactions is adapted, and which he himself influences. The human organism can directly represent elements at its own level, humans and other living beings, and more difficultly dead things. The human organism is also able to represent lower levels, such as organ systems, and higher levels, such as the family. A. Wholeness, intention and perception. B. The 5 representational stages: 1. Directly representation of the outer world. 2. Indirectly representation of the outer world through the brain. 3. Representation of the brain in the wholeness. 4. Directly representation of the outer world. 5. The wholeness represents the wholeness.

We believe that the brain can represent the wholeness of the organism and what goes on in it in addition to representing the outer world through the senses. This means that the organism possesses the possibility of the rational and the emotional interpretations of reality. When the simplistic representation of the world dominates in the representation of the wholeness we have the rational interpretation of reality.

When the direct representation outside the brain dominates the representation of the wholeness, we have the emotional interpretation of reality, where the brain represents the wholeness[3]. In this paper we will discuss man as a wholeness through his ability to experience the world through these two ways of interpretation.

## THE HUMAN AS A WHOLENESS

### Attention, Perception, and Consciousness

The information transmitting interactions giving representation of a subject matter in the wholeness of the organism corresponds to the attention function, and the representation itself corresponds to the attention. This is in contrast to the common notion that perception happens in the brain[5]. However we think that consciousness cannot be explained by brain activity alone. Consciousness is not brought about before the totality, the seat of perception, has represented the brain. Consciousness demands a representation of the subject matter in the totality of the organism, but this is not sufficient. A double, simultaneous representation is required, because the representation is a sufficient model of reality within the brain[3,6]; the comparison of the two representations is necessary for being able to witness the world, in other words: to be conscious about it.

## Thought, Fantasies, Visions, and Dreams

When the totality represents the brain in a state, where important new sensory information is not entering, but where the rational interpretation of reality is sustained, the purpose of the organism of handling the outer world manifests itself through a reorganization of the representation in the brain. The model of reality in the brain is reorganized in a way that makes it possible to retain a better handling of the world through behavior. When the brain is working with space-time organizations this phenomenon is called thinking, and when the pictures do not represent the real world it is called visions or fantasies. In dreams, the possibility of realizing the purposes of the organism under the existing circumstances is systematically worked through.

## The Intent

It is evident that representation is very tightly controlled through the perception function. The patterns of the totality are tuned in a way that ensures agreement between the patterns of the totality and the patterns of a specific level or subject. This tuning is the purpose of the organism. The tuning of the totality happens, when information-transmitting interactions bring the “programs” lying as organized patterns at the deepest levels to the surface of the wholeness of the organism.

The subject matter that is represented has a given organization, which by now can be evaluated through more specific interactions between the wholeness and the information-“program”. Reality is thus perceived and obtains its meaning through the purposes of the biological system. These purposes are a collection of fundamental biological information patterns forming the basis for the unfolding of the biological system. Various aspects of wholeness are submitted to each purpose, and correspondingly different aspects of reality are perceived as a consequence of this submission.

Attention may be attracted to incidents at the various levels, and the purposes must therefore be recruited dynamically as they are needed in order for the wholeness to undergo governed transformations in a suitable way. The governing of attention must happen autonomously through shifts between the states of the totality following a genetically determined governing pattern. The realization of the more simple patterns is a condition for the more complex organizations, in the same way as in morphogenesis[7,8].

## Morals and Ethics

What attention captures is perceived and gives meaning in relation to the basic pattern, the purpose. The organization of the subject matter may be viewed as proper or improper – or it may be viewed as something in between. When a level or a subject matter is represented in the totality, a double evaluation follows. On the surface the organization is perceived as suitable for the organism or unsuitable for the organism, i.e. right or wrong. Inwardly an emotional response is triggered, that evaluates the agreement between the genetic program and the structure of the subject matter in the representation of the totality. A

good agreement between the program and reality corresponds to positive emotions, while a bad agreement corresponds to negative emotions.

## The Will

Hitherto we have only been looking at the representation of subject matters in the totality of the organism through the attention function. It is secondary whether the representation happens directly through the totality following a purpose, resulting in an emotional interpretation of reality, or indirectly through the brain following a purpose, resulting in a rational interpretation of reality. The evaluation through emotion and perception happens in the same way.

Information transmitting interactions in principle work both ways. When purpose transmits information to the wholeness of the organism, information is also transmitted from this wholeness to the subject matter at which the attention is directed. A representation is forced upon the subject matter by the organism, and this function, which is the inverse of the attention function, is called the function of will.

The organism approaches the subject gradually. At first a fundamental adaptation is accomplished by the tuning of the totality by the purpose. In this way the basic patterns are shared between the organism and the subject. This creates a representation of the subject in the totality of the organism. Then this representation is evaluated, and the purpose is tightened, and the patterns corresponding to a certain organization of the subject are pulled up into the totality. Vice versa the desired organization is transferred to the subject.

The increasing complexity of the organism throughout life can be understood as the realization of purposes through the use of the will function. The different purposes are recruited in a succession that ensures organization of the inner levels before the outer levels. The will function is thus the organism's organizing force. It is brought about through information transmitting interactions. The purpose being a very high level of organization in the totality of the organism, forces an organization corresponding to the informational program of the organism, onto a subject with which the totality interacts. The will function can work directly upon the outer world, or indirectly through the brain. The latter leads to behavior, and in man also to speech. Speech is an indirect mediation of interpretation of reality through words, while action is a direct mediation of interpretation of reality through the motor apparatus. It is possible to act out of an emotional interpretation of reality, because the brain is subject to the totality in this interpretation of reality[3,6]. Direct use of the will function is usually hidden from our reason and belongs to the emotional interpretation of reality.

## The Intuition

The direct interaction (described above) either between individuals or between an individual and the society yields a complex information transmission, but this is only available for the emotional interpretation of reality. A specific representation of the outer world focusing at certain patterns may theoretically speaking be a precise source of information, providing that patterns that reach us through information transmitting interactions can be identified and interpreted in a precise and sober-minded way.

The difficulty of obtaining this precision and sober-mindedness has led to a pronounced and legitimate skepticism against the value of this data source, because people who claim to have intuition rarely have developed this gift. Intuition in its developed form is the ability to let one's attention float in the complex dynamics consisting of the wholeness and all the individuals of the society. When the wholeness represents the outer world in a general way, one obtains experiences of unity with nature and the like, that belongs to the class of experiences that collide with an undeveloped reason.

## The Collective (Un-)Consciousness

When you start thinking in this strange way, that reality is more a hidden order that what meets the eye; the reality of a collective mind or collective consciousness is not a farfetched idea. If the world is created like a huge many-leveled fractal, we might all be a part of such a huge meta-structure, which connects us and collects our perceptions and experiences to bring them to the next level for all to enjoy. This is in a way scary because we often prefer that our consciousness is private; the Jungian concept of a common consciousness is basically born our of the opposite idea: that consciousness cannot be private; it is always public although most people luckily for us do knot know how to approach the collective consciousness and "crack the code" to get admission to our innermost hidden secrets and unspoken emotions.

### The Religious Experience

Last we will discuss the religious experience. In our culture one of the least known qualities of man's wholeness is man's ability to represent himself, to be aware of himself. This is an experience obtained either by chance or, more commonly, after having exercised the control of attention to a level, where he can exclude all other representations. It is usually described as the religious experience: the experience of God, of the divine principle enlightenment etc. In spite of the evidently positive, beautiful and real aspects of this experience, it demands much soberness to be able to use this kind of experiences in a positive way. Very often people lose their way in the overwhelming experiences, or they use them for self-asserting purposes, a fact that has resulted in a prevailing and justified skepticism against "religious" people.

## CONCLUSIONS

Many aspects are involved when we look at the man as wholeness, including a suitable combination of the rational and emotional interpretation of reality. These aspects are summarized and discussed in this paper:

- We think that consciousness cannot be explained by brain activity alone. A double, simultaneous representation is required. The comparison of these two representations is necessary to be conscious about the world.
- The phenomenon of thinking is when the brain is working with space-time organizations leading to pictures. When these do not represent the real world it is called visions or fantasies. In dreams the possibility of realizing the purposes of the organism is systematically worked through.
- The patterns of the totality are tuned in a way that ensures agreement between the patterns of the totality and the patterns of a specific level or subject. This tuning is the organism's intent.
- The organization of a subject matter may be viewed as proper or improper or something in between. A good agreement between the genetic program and reality corresponds to positive emotions, while a poor agreement corresponds to negative emotions. Tuning of these functions is called moral and ethics.
- A representation is forced upon a subject matter by the organism. This function is called the function of will and can work directly upon the outer world, or indirectly through the brain. The latter leads to behavior, and in man also to speech. Speech is an indirect mediation of interpretation of reality through words, while action is a direct mediation of interpretation of reality through the motor apparatus.
- The direct interaction between individuals or between an individual and the social levels yields a complex information transmission available for the intuition. In its developed form intuition is the ability to let one's attention float in the complex dynamics consisting of the wholeness and all the individuals of the society.
- In our culture one of the least known qualities of man's wholeness is man's ability to represent himself, to be aware of himself. This is an experience obtained either by chance or after having exercised the control of attention to a level, where he can exclude all other representations. It is usually described as the religious experience.

## ACKNOWLEDGMENTS

These studies were supported by grants from IMK Almene Fond. Our research in quality of life has been approved by the Copenhagen Scientific Ethical Committee under number (KF)V.100.2123/91.
